# The complete chloroplast genome of *Prunus tangutica* (Batal.) Korsh

**DOI:** 10.1080/23802359.2021.1997110

**Published:** 2022-01-24

**Authors:** Jinfeng Niu, Qiang Zhu, Jijuan Zeng

**Affiliations:** a Ningxia State-owned Forest Farm and Forest Seedling Station, Yinchuan, Ningxia, China; b Ningxia Forestry Institute Co., Ltd, Yinchuan, Ningxia, China; c State Key Laboratory of Seeding Bioengineering, Yinchuan, Ningxia, China

**Keywords:** *Prunus tangutica*, chloroplast genome, phylogenetic analysis

## Abstract

The complete cp genome of *Prunus tangutica* is 158,131 bp in length, exhibits a typical quadripartite structural organization, consisting of a large single copy (LSC) region of 86,266 bp, two inverted repeats (IR) regions of 26,389 bp, and a small single copy (SSC) region of 19,087 bp. The cp genome contains 131 complete genes, including 86 protein-coding genes (86 PCGs), 8 ribosomal RNA genes (8 rRNAs), and 37 tRNA genes (37 tRNAs). Most genes occur in a single copy, while 19 genes occur in double, including 4 rRNAs (4.5S, 5S, 16S, and 23S rRNA), 7 tRNAs (*trnA-UGC*, *trnI-GAU, trnL-CAA*, *trnI-CAU, trnN-GUU*, *trnR-ACG*, and *trnV-GAC*), and 5 PCGs (*rps7*, *ndhB*, *ycf2*, *rpl2* and *rpl23*). The overall GC content of cp DNA is 36.7%, the corresponding values of the LSC, SSC, and IR regions are 34.6%, 30.1%, and 42.6%, respectively. Further, the phylogenetic analysis suggested that the *P. tangutica* was closely related to *Prunus tenella*. The results of *P. tangutica* will lay a foundation for further research.

## Introduction

*Prunus* L. belongs to the subfamily Amygdaloideae of the Rosaceae. It consists of 200 species with most species in the temperate zone (Yu et al. 1986; Ghora and Panigrahi 1995; Mabberley 1997). *Prunus tangutica* (Batal.) Korsh. is economically important because many species are sources of oil, timber, and ornamentals (Sangtae and Jun 2001). However, due to anthropogenic overexploitation and decreasing distributions, this species needs urgent conservation. Knowledge of the genetic information about this species would contribute to the formulation of a protection strategy. In this study, we assembled the complete chloroplast genome of *P. tangutica*, hoping to lay a foundation for further research.

Fresh leaves of *P. tangutica* were collected from the psammophyte germplasm bank of Yinchuan Botanical Garden (Yinchuan, Ningxia, China; coordinates: 105°49′18″E, 38°08′42″N) and dried with silica gel. The voucher specimen was stored in the State Key Laboratory of Seeding Bioengineering with the number is NFILSBZJ20210116. Plant Chloroplast Genomic DNA was extracted with a modified CTAB method (Doyle and Doyle 1987). We used Illumina HiSeq X Ten sequencing and MITObim v1.9 program of Hahn et al. (2013), with the help of close reference sequences to assemble chloroplast genomes. We assembled the complete chloroplast genome by GetOrganelle pipeline v1.6.3a (Jin et al. 2020). Plann v1.1 (Huang and Cronk 2015), Geneious v11.0.3 (Kearse et al. 2012) were used to annotate the chloroplasts genome and correct the annotation. The OGDraw online tool (Lohse et al. 2013) (https://chlorobox.mpimp-golm.mpg.de/OGDraw.html) was used to produce a genome map. The complete cp genome was deposited in GenBank (accession number: MZ145044)

The total plastome length of *P. tangutica* is 158,131 bp, exhibits a typical quadripartite structural organization, consisting of a large single copy (LSC) region of 86,266 bp, two inverted repeats (IR) regions of 26,389 bp, and a small single copy (SSC) region of 19,087 bp. The cp genome contains 131 complete genes, including 86 protein-coding genes (86 PCGs), 8 ribosomal RNA genes (8 rRNAs), and 37 tRNA genes (37 tRNAs). Most genes occur in a single copy, while 19 genes occur in double, including 4 rRNAs (4.5S, 5S, 16S, and 23S rRNA), 7 tRNAs (*trnA-UGC*, *trnI-GAU, trnL-CAA*, *trnI-CAU, trnN-GUU*, *trnR-ACG*, and *trnV-GAC*), and 5 PCGs (*rps7*, *ndhB*, *ycf2*, *rpl2* and *rpl23*). The overall GC content of cp DNA is 36.7%, the corresponding values of the LSC, SSC, and IR regions are 34.6%, 30.1%, and 42.6%.

**Figure 1.**
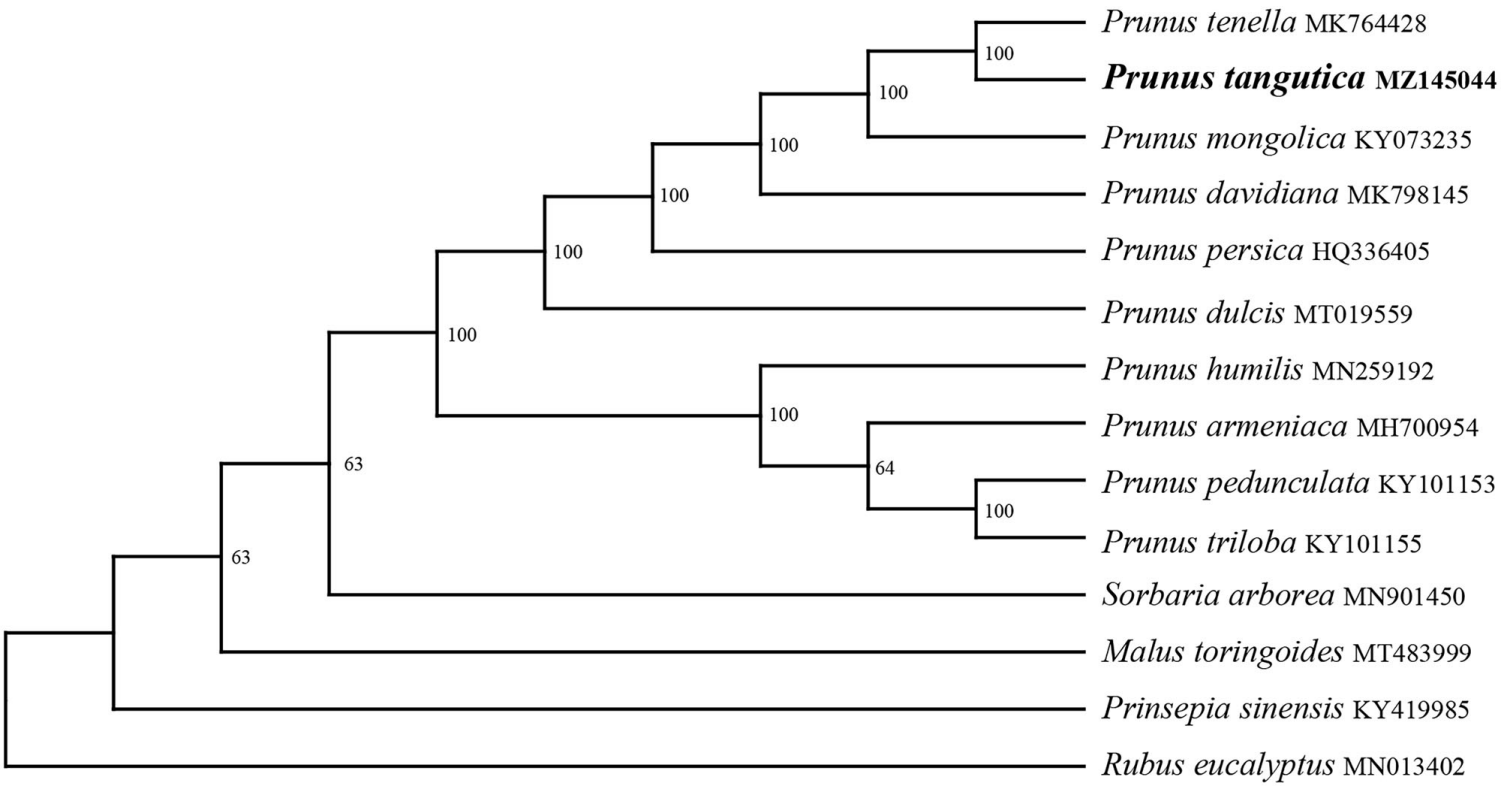
Maximum likelihood(ML) analysis of *Prunus tangutica* and other related species based on the chloroplast genome sequence.

In order to further clarify the phylogenetic position of *P. tangutica*, a plastome of 13 representatives *Prunus* species were obtained from NCBI to reconstruct the plastome phylogeny, with *Sorbaria arborea*, *Malus toringoides*, *Prinsepia sinensis,* and *Rubus eucalyptus* as an outgroup. All the sequences were aligned using MAFFT v.7.313 (Katoh and Standley 2013) and maximum likelihood phylogenetic analyses were conducted using RAxML v.8.2.11 under GTRCAT model with 500 bootstrap replicates. The phylogenetic tree shows that the species of *Prunus* were divided into two subclades (Figure 1). *P. humilis*, *P. armeniaca*, *P. pedunculata,* and *P. triloba* clustered together, and remain species clustered in another clade. while *P. tangutica* is a sister to *P. tenella*.

## Data Availability

The genome sequence data that obtained at this study are openly available in GenBank of NCBI (https://www.ncbi.nlm.nih.gov/) under accession number of MZ145044. The associated BioProject and SRA numbers are PRJNA762480 and SUB10364517, respectively.
